# Globalization of national surgical, obstetric and anesthesia plans: the critical link between health policy and action in global surgery

**DOI:** 10.1186/s12992-019-0531-5

**Published:** 2020-01-02

**Authors:** Paul Truché, Haitham Shoman, Ché L. Reddy, Desmond T. Jumbam, Joanna Ashby, Adelina Mazhiqi, Taylor Wurdeman, Emmanuel A. Ameh, Martin Smith, Edwin Lugazia, Emmanuel Makasa, Kee B. Park, John G. Meara

**Affiliations:** 1 Program in Global Surgery and Social Change, Harvard Medical School, Boston, MA USA; 2 Department of Plastic and Oral Surgery, Boston Children’s Hospital, Boston, MA USA; 3 Department of Surgery, National Hospital, Abuja, Nigeria; 4 Department of Surgery, University of the Witwatersrand, Johannesburg, South Africa; 5 Department of Anaesthesiology, Muhimbili University of Health and Allied Sciences (MUHAS), Dar es Salaam, Tanzania; 6 PSMD-Cabinet Office, Office of the President, Lusaka, Zambia; 7 Wits Centre of Surgical Care for Primary Health and Sustainable Development, University of the Witwatersrand, Johannesburg, South Africa

**Keywords:** Global surgery, Essential surgery, Universal health coverage, Global Health, Globalization, Noncommunicable diseases, Global Health systems, Health policy

## Abstract

Efforts from the developed world to improve surgical, anesthesia and obstetric care in low- and middle-income countries have evolved from a primarily volunteer mission trip model to a sustainable health system strengthening approach as private and public stakeholders recognize the enormous health toll and financial burden of surgical disease. The National Surgical, Obstetric and Anesthesia Plan (NSOAP) has been developed as a policy strategy for countries to address, in part, the health burden of diseases amenable to surgical care, but these plans have not developed in isolation. The NSOAP has become a phenomenon of globalization as a broad range of partners – individuals and institutions – help in both NSOAP formulation, implementation and financing. As the nexus between policy and action in the field of global surgery, the NSOAP reflects a special commitment by state actors to make progress on global goals such as Universal Health Coverage and the United Nations Sustainable Development Goals. This requires a continued global commitment involving genuine partnerships that embrace the collective strengths of both national and global actors to deliver sustained, safe and affordable high-quality surgical care for all poor, rural and marginalized people.

## Background

In 2015 the Lancet Commission on Global Surgery estimated that nearly 5 billion people lack access to safe, affordable and timely surgical and anesthesia care. Since then, efforts to expand access to surgical care through coordinated health policy efforts have substantially evolved. The National Surgical, Obstetric and Anesthesia Plan (NSOAP) emerged as a policy framework to systematically and comprehensively address the health burden of conditions requiring surgery. This paper highlights the need for a continued globalized approach through genuine partnerships that embrace the collective strengths of local and international organizations to deliver quality surgical, obstetric and anesthesia care for all.

### Academic global surgery: from individual mission to global health policy

Low and middle income countries (LMICs) have made significant progress towards improving healthcare by focusing on communicable diseases [1–3]. The early focus of global health on infectious diseases and vaccination campaigns led to increased life expectancy, but did not address non-communicable diseases such as cardiovascular disease, cancer and injury [4]. This was due, in part, to the perceived low disease burden of these NCDs compared to communicable diseases and the perceived high cost and complexity of surgical care including infrastructure, workforce and reliable supply chains. Currently, potential deaths averted by surgery and anesthesia (16.9 million in 2010) outnumber historical communicable disease targets including tuberculosis (1.2 million), HIV (1.46 million) and malaria (1.17 million) combined [5, 6]. As the need for safe, affordable, high-quality surgical, obstetric and anesthesia care has become more apparent, policy efforts have shifted to include this work in global health initiatives.

The movement towards a formal acknowledgement of surgical care within universal healthcare began in 1980 when the director-general of the World Health Organization (WHO), Dr. Halfdan Mahler, commented on the disparities in surgical care in his address to the International College of Surgeons in Mexico City [7]. At the time, his call for increased focus on surgical care in low-resource settings went largely unanswered. Nearly 30 years later, Dr. Paul Farmer and Dr. Jim Kim published *Surgery and Global Health: A view beyond the OR*, noting that surgery was the “neglected stepchild of global health” [8]. While surgeons continued to provide needed surgical care through efforts by local health workers, mission trips and education initiatives, surgery and anesthesia was not prioritized from a national or international policy, public health or health economics standpoint.

In 2015 three landmark publications catalyzed the global surgery and anesthesia movement. For the first time, a surgery-dedicated volume in the *Disease Control Priorities 3* (DCP-3) publication by the World Bank Group presented surgical care as cost-effective interventions to address the global disease burden [6]. The Lancet Commission on Global Surgery’s - *Global Surgery 2030: evidence and solutions for achieving health, welfare, and economic development* highlighted global surgery as a public health epidemic by quantifying the disease and economic burden resulting from diseases amenable to safe, affordable and timely surgical, obstetric, and anesthesia care [9]. The publication estimated a lost economic output of $12.3 trillion for low-income countries between 2015 and 2030 with a global cost of only $350 billion required to avert those losses. Additionally, the World Health Assembly resolution (WHA) 68.15: *Strengthening emergency and essential surgical care and anesthesia as a component of universal health coverage*, approved by all 194 Member States, provided a mandate to include access to safe, affordable high quality surgical and anesthesia care as an essential part of drive towards Universal Health Coverage. While the mandate from the WHO did not specify how the immense worldwide surgical burden would be addressed, it elevated global surgery into the public policy realm. In the year 2017, through Decision WHA70(22), Member States tasked the WHO to implement resolution WHA68.15 as part of the organization’s work on SDGs and report on progress bi-annually till the year 2030 while this year, 2019, the new first African WHO Director-General, Dr. Tedros Adhanom, stressed the importance of surgical care in the global and country work to attaining universal health coverage at the partnership meeting under the theme “*National Surgical, Obstetric, and Anesthesia High-Level Planning Meeting for Global, Regional, and Country Authorities and Funders”* in Dubai, United Arab Emirates [10].

Efforts towards improving surgical, anesthesia and obstetric care have evolved from a primarily short-term ‘mission trip’ model of volunteer surgical delivery in low resource settings to support the efforts of local healthcare workers to a more sustainable health system strengthening approach. This has precipitated a new field of “academic global surgery” that envisions a multidisciplinary, evidence-based, health equity approach to surgical care in low-resource settings. This includes teams of policy experts, physician-researchers, economists, local and federal governments, industry partners, professional societies, and advocacy groups. The globalized world is one defined by its “networks of interdependence” [11]: challenges are shared and solutions require a regional or global approach. Academic institutions work to ensure that mission trips are focused on sustainable capacity building by combining service, skills transfer, and education. Countries have organized large scale, ministry of health-led efforts to develop context-specific NSOAPs as a policy guide to finance and improve access to high-quality and affordable surgical care. As LMICs strive to improve their surgical health systems, it has become clear that a national approach, factoring in the complexities of globalization, is required to shape surgical, obstetric and anesthesia care service delivery.

### NSOAP: global surgery policy in action

**Fig. 1:**
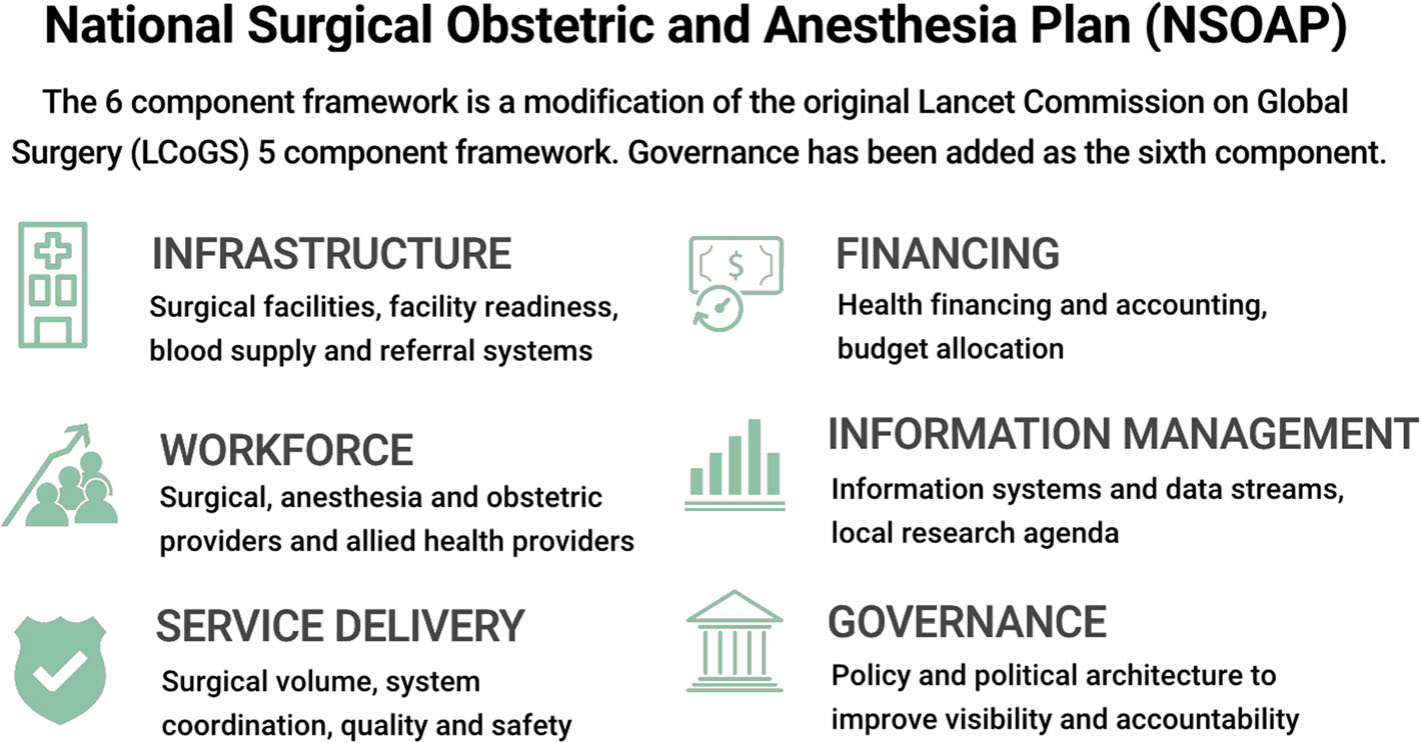
NSOAP development includes a complex framework of components that are often developed in parallel. These are not prescriptive, but flexible and adaptable to the local context of each countryGlobal Distribution of NSOAP Plans

**Fig. 2:**
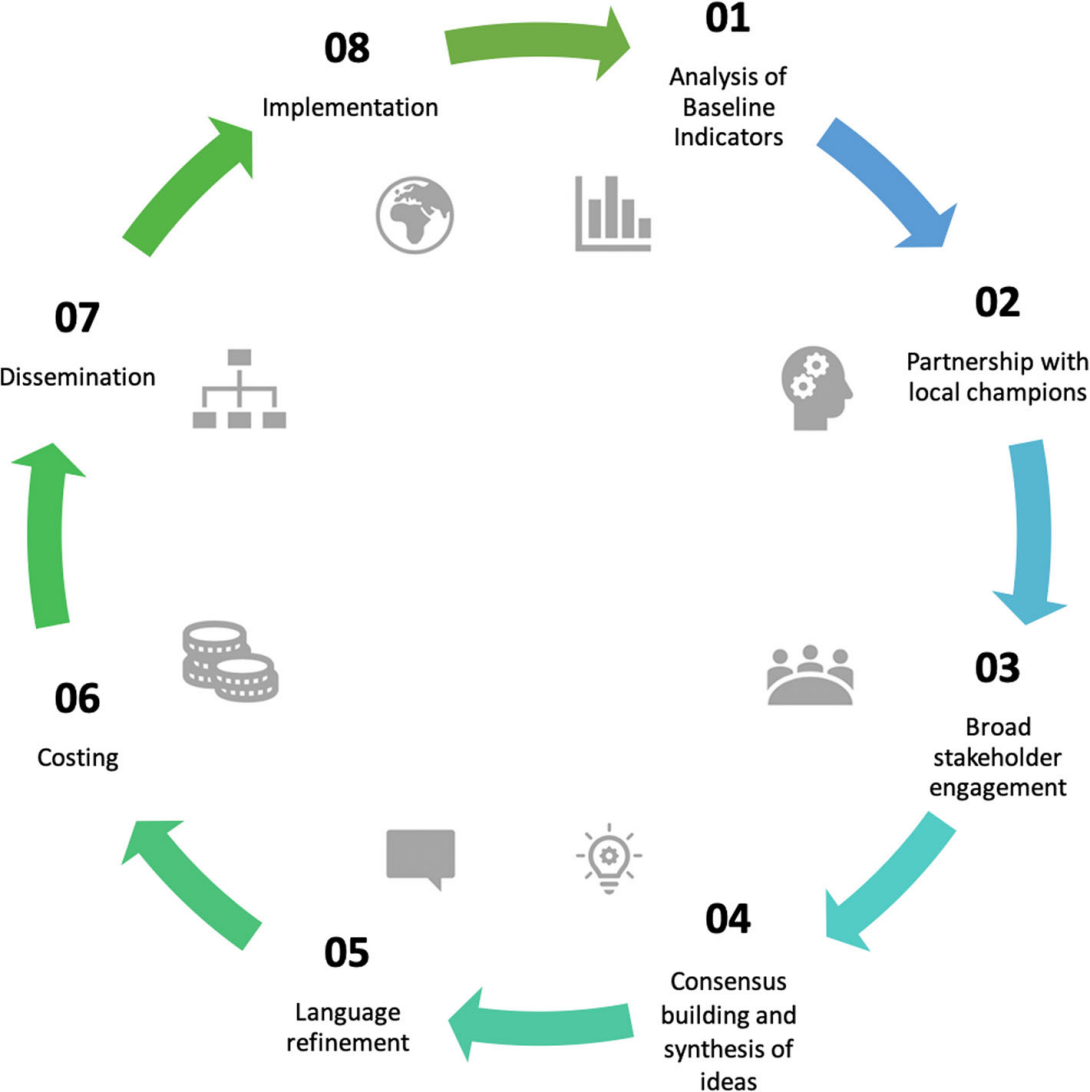
Eight stages of NSOAP development

Strengthening surgical, obstetric, and anesthesia care delivery requires both a functional and resilient health system as well as support from regional and global stakeholders [9, 12]. Despite the significant global burden of surgical conditions, surgery and anesthesia remains poorly represented in many national health policies and strategies [13]. Working towards universal access to safe, timely and affordable surgical, obstetric and anesthesia care, the Lancet Commission on Global Surgery (LCoGS) introduced a framework for national surgical, obstetric and anesthesia planning (NSOAP) (Fig. 1) [9]. The framework offers a systematic approach to strengthen surgical systems, covering six domains of the health system: infrastructure, service delivery, surgical workforce, information management, financing, and governance [12]. To develop an NSOAP, eight stages have been suggested (Fig. 2). The NSOAP framework and process addresses three core concepts inherent in any type of strategic planning exercise; defining current gaps in surgical care access and delivery, prioritizing solutions and setting targets, and providing a costed implementation framework together with a monitoring and evaluation plan [12].

Translation of academic research into an actionable process requires a concrete and contextualized implementation strategy led by relevant stakeholders including local champions, frontline providers, government leadership and regional coordination throughout the process [14]. Based on the NSOAP formulation processes adopted by different countries thus far, both centralized (e.g. Zambia and Tanzania) and decentralized (e.g. Pakistan) models have emerged as potential models for developing the NSOAPs [15]. These models embrace the local governmental institutional setup and prioritize the role of local champions, ministries of health, and regional partners in the coordinated effort of national surgical planning.

The key distinction between the centralized and decentralized model is the level at which priorities are set. In a centralized model, the Ministry of Health sets the priorities of the NSOAP and possesses the authority to implement the plan. The Ministry of Health works closely with local stakeholders to gain consensus from all key stakeholders including, frontline providers, governmental, non-governmental bodies, academic institutions and the private sector. The Ministry of Health leads the coordination of information gathering, conducts needs assessments, and works to develop a formal NSOAP that will be adopted and launched. The Ministry of Health, in coordination with all these agencies, develops a plan that aligns with the governmental priorities to be integrated into the country’s long-term national health policy strategic plan. Countries that have pursued a centralized model include Zambia, Tanzania, Ethiopia, Nigeria and Rwanda.

In a decentralized model, authority is shared between the national Ministry of Health and its state/provincial government thus allowing shared responsibility in the provision of preventive and curative services. A decentralized model fits countries with a devolved health system where the national Ministry of Health has laid out a broad national health policy framework or vision, and each state/province/governorate is granted the authority to implement a plan based on national priorities. The Ministry of Health sets national guidelines, oversees health regulation, national disease surveillance and provides a template for each state that can be used and coordinates and liaises efforts with other stakeholders nationally and internationally. Pakistan is an example of a country that has embarked on an NSOAP process using the decentralized model where the NSOAP has been adopted and modified as the high-level National Vision for Surgical Care 2025. Each province is then tasked to develop a provincial surgical, obstetric and anesthesia plan (PSOAP) to ensure success within each local context.

As countries develop national strategies for addressing their surgical burden, regional bodies have stepped up to support both centralized and decentralized national governance approaches to improve surgical care. In Africa, the Southern Africa Development Community (SADC) ratified a resolution to prioritize surgical care as part of its regional health strategy [16]. SADC is an intergovernmental organization that comprises sixteen Member States in Africa, 345 million people, and a collective GDP of $721.3 billion (USD) [17]. SADC is a regional economic zone of the African Union, which fosters cooperation, and integration towards common regional goals for sustainable development, economic growth, and peace. Regional health plays a central role in helping to mediate these shared objectives and is a necessary component of enhancing human capital for equitable and sustainable development. In 2018 a resolution was ratified at the annual senior officials and health ministers Conference, formally recognizing the role of surgical care in attaining regional development goals. This recognition is important as regional economic entities may be strategically influential for global surgical scale-up. Since Ministries of Finance are central players that influence decision-making at regional bodies, resolutions such as the SADC resolution and the ECSA-HC resolution on SOA could help to establish an enabling environment for financing and implementation of national programs to improve surgical care as part of universal health coverage.

Similarly, the Health Ministers of the Pacific region have prioritized safe and affordable surgical care for the region during the Pacific Health Ministers Meeting last August. In response, at the last Regional Committee Meeting (RCM) in October 2019, the Member States of the Western Pacific Region of the WHO recommended adding safe and affordable surgical care to the next RCM in Kobe, Japan in 2020. Assuming the Executive Board of the WHO approves the RCM agenda as is, the appearance of surgery on the RCM agenda would be pivotal in increasing dedicated funding for staff and programming within the WHO Regional Office and opportunities for the Member States to report on their progress in improving access to safe and affordable surgical care in their countries.

These strategic approaches are not meant to be prescriptive; instead, they are meant to guide the formulation process by providing the forum and space for consensus around NSOAP content and possible models for implementation. Although the WHA Resolution 68.15 and the WHA Decision 70.22 call upon each Member States to strengthen emergency and essential surgical care and to report on the progress every 2 years until 2030, the buy-in from ministries of health may require evidence-based and data-driven arguments on the unmet need for surgical care within an individual country [18]. The proposed NSOAP framework contributes to the achievement of SDG 3 in efforts to support achievement of universal health coverage. In addition, NSOAPs are linked to other SDGs including 1, 3, 5, 8, 9, 10, 16 and 17 [19]. Ultimately, making a case for NSOAPs involves presenting national planning as a coordinated and cost-effective effort to systematically improve surgical, obstetric and anesthesia care.

### Partners in a globalized context: from individuals to institutions

The dependency of safe surgical, obstetric, and anesthesia care on physical infrastructure, especially in contexts of resource scarcity, necessitates a regional approach. Countries working towards developing robust surgical care infrastructure can obtain assistance from multiple sources including individual experts, development banks, global professional societies and the WHO. Collectively, a broad range of individuals and institutions help in both the NSOAP formulation and implementation processes. Additional support from funding these efforts represent formal governmental policy planning and acknowledge NSOAPs as a coordinated and systematic framework, working towards universal access to safe, timely and affordable surgical, obstetric and anesthesia care.

Perhaps most importantly, local experts and champions have emerged who are able to provide expertise, support and guidance for regional and international national planning. These local experts possess the combined knowledge of local policy, customs and needs as well as the technical knowledge and relationship within the development community to provide longitudinal support for efforts.

Additionally, the WHO provides technical assistance to Member States for the development of national health plans through its country and regional offices and headquarters. Furthermore, the WHO plays a central role in the integration of surgical care programming across the entire ‘health system strengthening paradigm’ through its Global Initiative for Emergency and Essential Surgical Care (GIEESC), established in 2005 to convene multidisciplinary stakeholders in public health, government and international organizations [20].

International stakeholders such as the International College of Surgeons (ICS), the World Federation of Societies of Anesthesiologists (WFSA), the World Federation of Neurosurgical Societies (WFNS), and the International Federation of Gynecology and Obstetrics (FIGO) also fill a critical advocacy role in elevating surgical, obstetric, and anesthesia care within the global health agenda. A number of academic institutions across the world have taken an active role through leading longitudinal research endeavors, establishing bidirectional collaboration and promoting policy related work.

### Successes and challenges: the need for a globalized approach

**Fig. 3:**
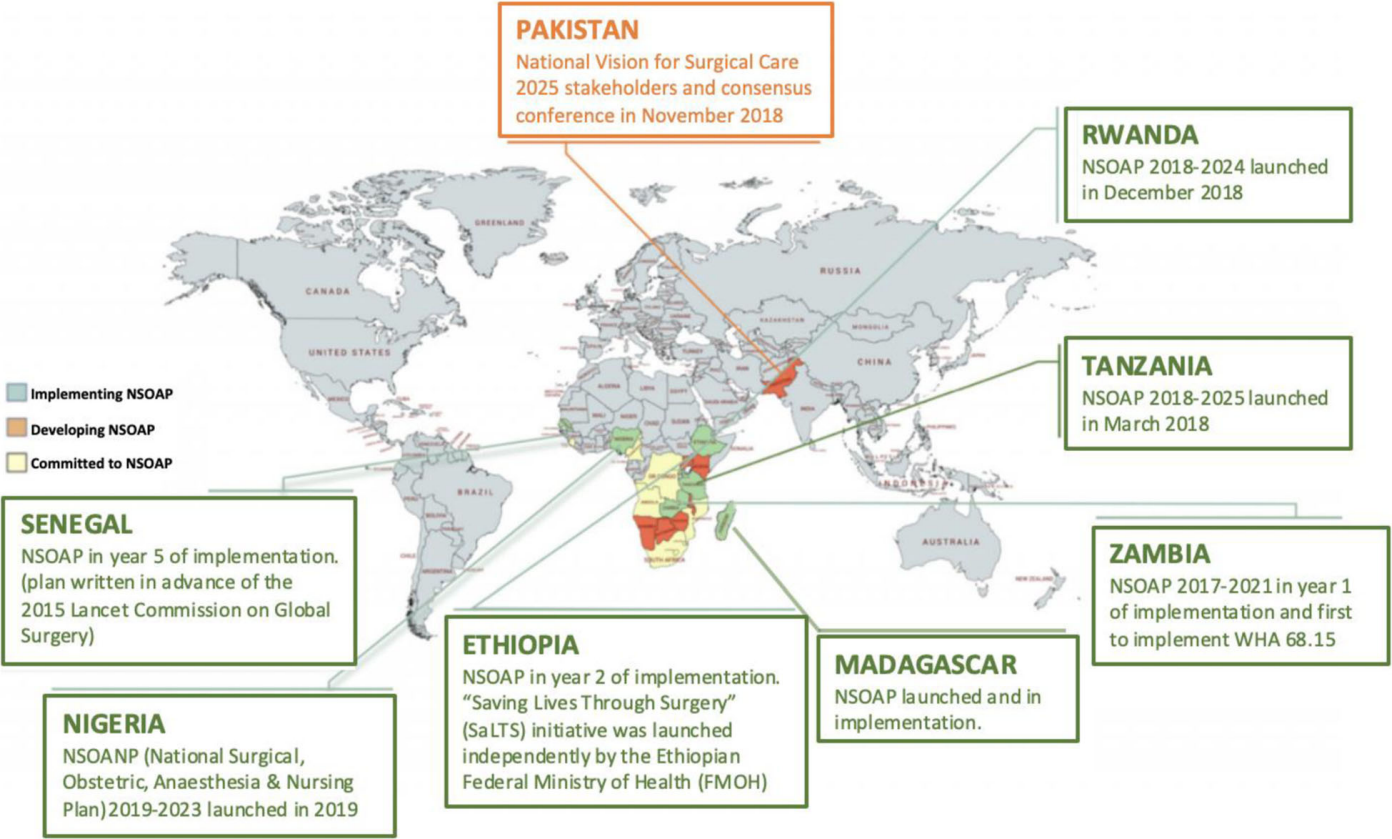
Global distribution of national surgical, obstetric and anesthesia plans (NSOAPs). NSOAPs are currently in various stages around the global ranging from commitment to implementation. Longitudinal monitoring, sustained political support, and financing will be necessary to ensure thatthese plans result in actionable improvement in access and quality of surgical care

To date, six countries have developed and launched NSOAPs. Over the past 2 years, Zambia, Nigeria, Madagascar, Rwanda and Tanzania have completed plans through the ministry of health-led approaches [21, 22] (Fig. 3). Senegal began their national plan prior to 2015 and is currently in the fifth year of implementation. Ethiopia independently adopted a national surgical plan ‘Saving Lives through Safe Surgery’ (SaLTS), Ten additional plans are underway, with a future 23 countries who have expressed commitment to the development of a NSOAP (Fig. 1) [12]. Latin American countries have begun plans for national surgical planning with a forum scheduled in early 2020 to bring together countries with interests on regionalizing the NSOAP model for South America [23]. Three key strategies are critical to achieving wider adoption of the NSOAP implementation process. These include data systems for indicator collection, financing and regionalization [18]. Unfortunately, there are currently limited efforts underway to ensure that surgical care policies are evidence-based and that interventions are cost-effective and clinically beneficial.

Indicator collection capacity can be improved through harnessing the collective strengths of partnerships with academic institutions, NGOs and private partners at the local and global level. Increased surgical volume without improved quality will result in significant mortality and necessitates high quality research to inform policies and indicators embedded within NSOAPst [13, 19]. Implementation research, a rapidly emerging field, has been described as “the scientific study of methods to promote the uptake of research findings and other evidence-based practices into routine practice, and, hence to improve the quality and effectiveness of health services and care.” [24] Methods of implementation science could be used to assess NSOAP development processes and factors related to successful or unsuccessful implementation. Additionally, funding for clinical trials such as those currently being conducted by the Globalsurg Collaborative and the NIHR Global Health Research Unit on Global Surgery will help ensure that improvement of clinical practice goes hand in hand with policy development and surgical scale up. Such research will inform future NSOAP development and implementation efforts to ensure that the best evidence-based practices are used [14].

Funding for NSOAPs also presents a unique challenge in LMICs requiring resource mobilization through both local and global actors. The LCoGS estimates that LMICs could lose up to 2% of their economic growth by 2030 through failure to establish surgical care systems, but individual countries struggle to develop a country specific estimate. To date, no country with an NSOAP has committed a significant budget for surgical care. At the country level, professional societies, academic institutions, media and citizens can play a critical role in advocating for increased budget allocation for NSOAP implementation. International funding is also needed for NSOAP implementation. A significant proportion of health care funding in LMICs is currently derived from external funders. For example, external funding accounted for 34% of Total Health Expenditure (THE) in Zambia in 2013 and 48% in 2011/2012 [25]. Mobilizing domestic funding would enhance the sustainability and accountability of these plans. Countries should explore context-specific ways of achieving this such as linking NSOAP implementation strategies to ongoing interventions and studies. This could allow flexibility in recasting the current budgets to cover NSOAP initial integration into national healthcare frameworks.

Greater efforts to globalize surgical system reform could help countries develop coherent strategies and agreements that better integrate and coordinate country plans to solve these shared areas of apprehension. Regional efforts should ideally be supported by a broad range of institutions, spanning the full spectrum of global health actors involved at both the domestic and regional levels. Inter-governmental bodies like the East African Community (EAC), for example, can liaise with WHO regional offices to reinforce country efforts through resource mobilization, monitoring progress and helping Member States to share knowledge and lessons learnt. A regional approach should be systematically studied such that these actors, together with Member States, are equipped with the necessary set of knowledge, concepts and ideas through which to better understand and implement the integration of NSOAPs at the inter-governmental level.

## Conclusion

Successful implementation of national surgical, obstetric and anesthesia plans in multiple countries has led to initial celebration, but true success will be the result of longitudinal monitoring, quality improvement and sustained political support and financing. The national surgical plan is the link between policy and action in the field of global health by reflecting a true commitment to improving outcomes for the 5 billion who lack access to safe, affordable surgery, obstetric and anesthesia care. This will require continued globalization through genuine partnerships that embrace the collective strengths of both local stakeholders and international organizations to deliver quality surgical care for all poor, rural and marginalized people.

## Data Availability

Not applicable.

## Abbreviations

AIDS: Auto-immune deficiency syndrome
ASEAN: Association of South-East Asian Nations
DCP-3: Disease Control Priorities – 3
EAC: East Asian Communities
GDP: Gross Domestic Product
GFF: Global Financing Facility
HIC: High Income Country
HIV: Human Immunodeficiency Virus
LCoGS: Lancet Commission on Global Surgery
LMICs: Low and Middle Income Countries
MoH: Ministry of Health
NSOAPs: National Surgical, Obstetric and Anesthesia Plans
RSOAP: Regional Surgical, Obstetric and Anesthesia Plans
SADC: Southern Africa Development Community
SaLTS: Saving Lives Through Safe surgery
SOA: Surgical, Obstetric and Anesthesia
THE: Total Household Expenditure
WHA: World Health Assembly
WHO: World Health Organization
